# Glucosinolates of *Sisymbrium officinale* and *S. orientale*

**DOI:** 10.3390/molecules27238431

**Published:** 2022-12-02

**Authors:** Azra Đulović, Marijana Popović, Franko Burčul, Vedrana Čikeš Čulić, Sandra Marijan, Mirko Ruščić, Nikolina Anđelković, Ivica Blažević

**Affiliations:** 1 Department of Organic Chemistry, Faculty of Chemistry and Technology, University of Split, Ruđera Boškovića 35, 21000 Split, Croatia; 2 Department of Analytical Chemistry, Faculty of Chemistry and Technology, University of Split, Ruđera Boškovića 35, 21000 Split, Croatia; 3 School of Medicine, University of Split, Šoltanska 2, 21000 Split, Croatia; 4 Department of Biology, Faculty of Science, University of Split, Ruđera Boškovića 33, 21000 Split, Croatia

**Keywords:** *Sisymbrium officinale*, *Sisymbrium orientale*, glucosinolates, desulfoglucosinolates, isopropyl isothiocyanate, antiproliferative activity

## Abstract

Glucosinolates (GSLs) from *Sysimbrium officinale* and *S. orientale* were analyzed qualitatively and quantitatively by their desulfo-counterparts using UHPLC-DAD-MS/MS. Eight GSLs were identified in *S. officinale*, including Val-derived (glucoputranjivin) and Trp-derived (4-hydroxyglucobrassicin, glucobrassicin, 4-methoxyglucobrassicin, and neoglucobrassicin) as the major ones followed by Leu-derived (Isobutyl GSL), Ile-derived (glucocochlearin) and Phe/Tyr-derived (glucosinalbin). Different *S. orientale* plant parts contained six GSLs, with Met-derived (progoitrin, epiprogoitrin, and gluconapin) and homoPhe-derived (gluconasturtiin) as the major ones, followed by glucosinalbin and neoglucobrassicin. GSL breakdown products obtained by hydrodistillation (HD) and microwave-assisted distillation from *S. officinale*, as well as isopropyl isothiocyanate, as the major volatile in both isolates, were tested for their cytotoxic activity using a 3-(4,5-dimethylthiazol-2-yl)-2,5-diphenyltetrazolium bromide (MTT) assay. Generally, all volatile isolates showed similar activity toward the three cancer cell lines. The best activity was shown by isopropyl isothiocyanate at a concentration of 100 µg/mL after 72 h of incubation, with 53.18% for MDA-MB-231, 56.61% for A549, and 60.02% for the T24 cell line.

## 1. Introduction

Glucosinolates (GSLs) are sulfur- and nitrogen-containing plant-specialized metabolites found in crucifers. Through their degradation products, mostly isothiocyanates, they are recognized as cancer prevention agents and biopesticides and are responsible for distinctive flavors. To date, 90 of 139 GSLs found in plant kingdoms have been fully characterized by modern spectroscopy techniques [1,2,3]. The structural diversity of GSLs is due to variation in chain-elongation and addition of methylene groups, and secondary modification (hydroxylation, methylation, oxidation, or desaturation in most cases) in the biosynthetic pathway of different amino acid precursors. Aliphatic GSLs represent the largest group derived mostly from methionine, while branched GSLs derived from isoleucine, leucine, or valine are more scarce. Arylaliphatic GSLs are derived from phenylalanine or tyrosine and indole GSLs from tryptophan, while some still have uncertain precursors [1]. The biosynthesis of GSLs has been well studied in *Arabidopsis* [4,5], *Brassica rapa* [6], and *B. oleracea* [7]. Aromatic GSL biosynthesis is less studied compared to aliphatic and indolic GSLs, which featured most prominently in the literature to date [5,8]. In addition, all knowledge gathered about GSL biosynthesis derives from homology with *Arabidopsis* and pertains to only a small percentage of the GSLs discovered. Therefore, data about the genes responsible for the biosynthesis of ‘exotic’ GSLs, such as (*R*)-4-(cystein-*S*-yl)butyl GSL (glucorucolamine), 3-methoxybenzyl GSL (glucolimnanthin), or others are completely absent from the literature [8].

The genus *Sisymbrium* (Brassicaceae family) comprises ca. 94 species disjunctly distributed in the Old (41 spp.) and the New World (53 spp.), among which 9 spp. are known to be wild-growing in Croatia [9,10]. The center of *Sisymbrium* diversity is the Irano-Turanian region [11]. The migration of *Sisymbrium* from the western Irano-Turanian floristic region to the Mediterranean coincides with the massive desiccation of the Mediterranean Sea, which started in the middle Miocene (13.0 million years ago) and ended with the beginning of the Pleistocene (around 5.33 million years ago). This resulted in a higher diversification occurrence of the Mediterranean *Sisymbrium* species. A clade consisting of Mediterranean *Sisymbrium damascenum*, *S. orientale*, *S. macroloma*, and *S. volgense* points toward a common ancestor of these species with *S. officinale,* which emerged much earlier (in the middle Pliocene) [11].

Early qualitative investigation of the GSL profile of *S. officinale* (hedge mustard, erysimum, or the singers’ plant) was performed indirectly by their degradation products, and the presence of isopropyl GSL (glucoputranjivin, **56**), *sec*-butyl GSL (glucocochlearin, **61**), 2-hydroxypent-4-enyl GSL (gluconapoleiferin, **38*S***), prop-2-enyl GSL (sinigrin, **107**), 4-hydroxybenzyl GSL (glucosinalbin, **23**) was suggested. HPLC analysis of desulfoglucosinolates from *S. officinale* confirmed the presence of **56** as the major GSL [12,13], which was later isolated from seeds and confirmed by NMR [14]. Additionally, Griffiths et al., also revealed indol-3-ylmethyl GSL (glucobrassicin, **43**) [12]. The GSLs of *S. officinale* wild-growing in Croatia were identified indirectly by their volatiles isolated by hydrodistillation from aerial parts and **56**, **61**, benzyl GSL (glucotropaeolin, **11**) and 2-phenylethyl GSL (gluconasturtiin, **105**) [15]. Studies of *S. orientale* are scarce, including only indirect analysis of GSL degradation products, suggesting the presence of 3-(methylsulfanyl)propyl GSL (glucoibervirin, **95**), but-3-enyl GSL (gluconapin, **12**), 2-hydroxybut-3-enyl GSL (progoitrin, **24*R*** or epiprogoitrin, **24*S***), **38*S***, **11**, **23**, and **105** [16,17]. There are several reports of GSLs from other *Sisymbrium* spp., which were all detected indirectly as well [18].

The aim of this study was to identify and quantify GSLs from wild-growing *S. officinale* and *S. orientale* in different plant parts by their desulfo-counterparts using UHPLC-DAD-MS/MS. Furthermore, *S. officinale* volatiles produced after hydrodistillation and microwave-assisted distillation were analyzed using GC-MS. The antiproliferative activity of these isolates and isopropyl isothiocyanate was investigated using the 3-(4,5-dimethylthiazol-2-yl)-2,5-diphenyltetrazolium bromide (MTT) assay against three human cancer cell lines (lung A549, bladder T24 and breast MDA-MB-231).

## 2. Results and Discussion

### 2.1. Glucosinolates and Volatile Constituents

**Figure 1:**
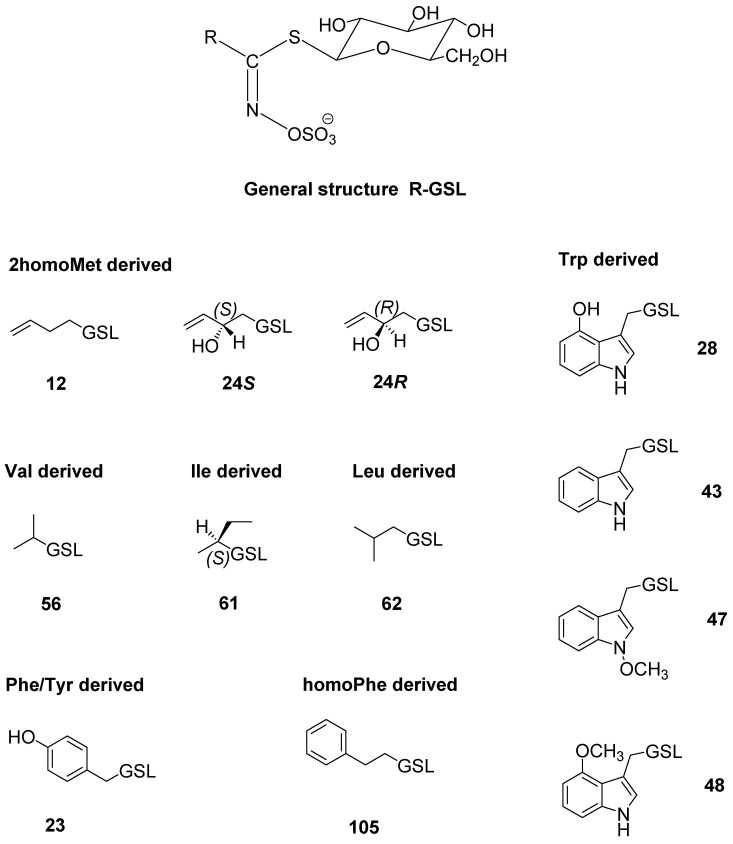
Structures of the GSLs identified in the investigated *Sisymbrium* sp. (cf. Table 1, Table 2 and Table 3).

The GSLs of *Sisymbrium officinale* and *S. orientale* were qualitatively and quantitatively analyzed using UHPLC-DAD-MS/MS by their desulfo counterparts. The GSLs detected in *S. officinale* are given in Table 1 and Table 2, and the ones found in *S. orientale* are given in Table 3. The structures of the corresponding GSLs are shown in Figure 1. The obtained chromatograms, as well as MS^2^ spectra of the obtained desulfoGSLs, are given in Figures S1–S4.

In total, eight GSLs were detected in *S. officinale*. An aromatic GSL, glucosinalbin (**23**), and indole GSLs, namely glucobrassicin (**43**), 4-hydroxyglucobrassicin (**28**), 4-methoxyglucobrassicin (**48**), and N-methoxyglucobrassicin (**47**), were identified by direct comparison to standards. Additionally, the important fragments being Na^+^ adduct ions used to identify and recognize all desulfoGSLs detected are annotated in more detail in the Supplementary data (Figure S4). Usual fragmentation of the thioglucosidic bond produces thioGlc fragment *m/z* 219 (type b), while the cleavage on the other side leads to [anhydroGlc + Na]^+^ at *m/z* 185 (type a) and type c fragment arising from loss of an anhydroGlc, [M-162 + Na]^+^ [19]. The characteristic loss of OCH_3_ radical from *N*-methoxy indole GSL of **d47** (*m/z* 390), as well as the loss of formaldehyde (*m/z* 391) can be observed in difference to C-methoxy indole GSL (**d48**) (Figure S4). Isopropyl isothiocyanate, the breakdown product of glucoputranjivin (**56**), was detected as the major volatile after thermal degradation in volatile samples obtained by hydrodistillation, as well as after microwave-assisted distillation, and was confirmed by direct comparison to standard as well. Isobutyl GSL (**62**) and *sec*-butyl GSL (**61**) were detected with MS^2^ spectra of the corresponding desulfoglucosinolates, which are identical (Figure S4) but have different retention times, *t*_R_ = 5.30 min and *t*_R_ = 5.64 min, respectively, which is in accordance with literature data [19]. Although limited, the identification of GSLs via the MS spectra of appropriate degradation products was very helpful in this case. Isobutyl isothiocyanate and *sec*-butyl isothiocyanate, having the same M^+^ (115) can be easily distinguished, as *sec*-butyl isothiocyanate produces characteristic fragment *m/z* 86 which is absent in the case of isobutyl isothiocyanate (not detected in the obtained volatile isolates, Table 2) [1].

Generally, isopropyl GSL (**56**), originating from Val biosynthesis, was the major GSL in all plant parts of *S. officinale*, ranging from 1.60 to 13.54 µmol/g DW in the Split sample and from traces to 7.40 µmol/g DW in the Krka sample. Other branched GSLs, such as isobutyl GSL (**62**), derived from Leu biosynthesis, and *sec*-butyl GSL (**62**), derived from Ile biosynthesis, were detected as minor GSLs.

Indole GSLs were detected with glucobrassicin (**43**) as the major one, ranging from 0.36 to 5.72 μmol/g DW. It was previously the only indole GSL detected in this plant, as far as the authors know [12]. In this study, three additional indole-type GSLs, 4-hydroxy-indol-3-ylmethyl GSL (**28**), 4-methoxyindol-3-ylmethyl GSL (**48**), and N-methoxyindol-3-ylmethyl GSL (**47**), were identified for the first time.

GSL **43** and its derivatives are usually found in many Brassica plants but not as dominant ones. Key enzymes for the modification of **43** are cytochrome P450 monooxygenases of the CYP81F subfamily and indole glucosinolate *O*-methyltransferases (IGMTs) of the plant *O*-methyltransferase family 2. CYP81Fs carry out hydroxylation reactions either in position 4 or 1 of the indole ring of **43**, and IGMTs convert hydroxy to methoxy groups [20,21,22]. CYP81F1 to CYP81F3 are capable of converting **43** to **28**, which is further metabolized by *O*-methyltransferases (IGMT1 to IGMT4) to **48**. On the other hand, CYP81F4 is capable of hydroxylating nitrogen (N) at position 1. This GSL was not detected. It is known that unsubstituted *N*-hydroxyindoles tend to be highly reactive, and stabilization is achieved by the conversion of the *N-*hydroxy group into an *N*-methoxy group, which is catalyzed by IGMT5 [23,24,25].

Glucosinalbin (**23**), the only arylaliphatic GSL, was identified in the root part of the Split sample. It can be suggested that this GSL originates directly from Tyr biosynthesis, although it can also be biosynthesized indirectly by hydroxylation of glucotropaeolin, which originates from Phe biosynthesis. However, glucotropaeolin was not identified.

In total, six GSLs were detected in *S. orientale* using the standards. The main GSLs in *S. orientale* were derived from Met biosynthesis (Table 3). According to the known biosynthesis of model plant *Arabidopsis thaliana*, Met is elongated into 2homoMet in the first biosynthetic step, after which 4-(methylsulfanyl)propyl GSL (glucoibervirin) is formed in the core structure biosynthesis pathway. The side chain of this GSL is then oxidized by flavin-containing monooxygenase, forming 4-(methylsulfinyl)propyl GSL (glucoiberin). These GSLs were not identified in the plant tissues. Conversion of the latter is regulated by the AOP2 genes, resulting in the identified but-3-enyl GSL (**12**). Hydroxylation of **12**, regulated by GS-OH genes, leads to progoitrin (**24*R***) and the main detected GSL epiprogoitrin (**24*S***).

*Sisymbrium orientale* also contained two arylaliphatic GSLs. Gluconasturtiin (**105**), originating from homoPhe biosynthesis and GSL **23**, was found only in traces in roots, which can originate from Phe or Tyr biosynthesis, as previously reported.

### 2.2. Antiproliferative Activity

The antiproliferative activity of the volatile isolates obtained from *S. officinale* by hydrodistillation (HD) and microwave-assisted distillation (MAD), as well as isopropyl isotiocyanate, their major volatile, were tested against three human cancer cell lines (lung A549, bladder T24, and breast MDA-MB-231).

**Figure 2:**
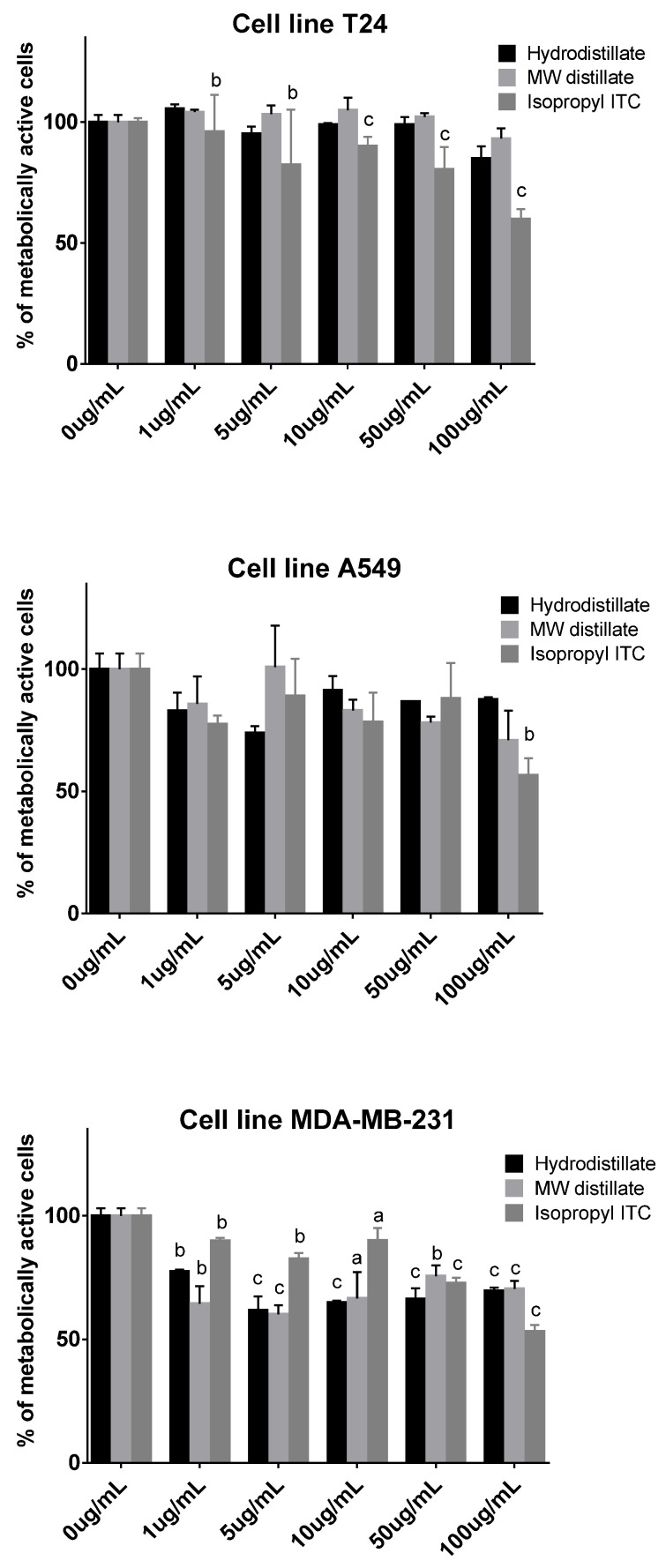
Percentage of metabolically active human lung cell lines A549, human bladder cell line T24, and human breast cell line MDA-MB-231 for different concentrations of *S. officinale* hydrodistillation and microwave-assisted distillation, as well as isopropyl isothiocyanate. Each data point is presented as the mean ± SD (*n* = 3). Lower case letters represent significance level in comparison to non-treated cell line samples (a, *p* < 0.05; b, *p* < 0.01; c, *p* < 0.001).

The best cytotoxic effect on all three cell lines was achieved after 72 h of incubation, but there was no difference in the action of the samples obtained using different isolation methods (Figure 2). The effectiveness of samples obtained from *S. officinale* and isopropyl isothiocyanate did not always correspond to the increase in concentration and incubation time and, in some cases, cell recovery occurred. Isopropyl isothiocyanate had a cytotoxic effect on all cell lines at a maximum concentration of 100 µg/mL after 72 h of incubation, with 53.18% for MDA-MB-231, 56.61% for A549, and 60.02% for the T24 cell line. A previous report of *Lunnaria annua* HD isolate, having 92% of isopropyl ITC, did not reach IC_50_ at 100 µg/mL for MDA-MB-231 and A549 cell lines tested, which is in accordance with this study [26]. Di Sotto et al. reported the antimutagenic activity of *S. officinale* extract, **56**, and isopropyl isothiocyanate against direct-acting (methyl methanesulfonate) and indirect-acting mutagens (2-aminoanthracene and 2-aminofluorene) in various *Escherichia coli* strains. The latter two are considered pro-carcinogenic compounds [27].

## 3. Materials and Methods

### 3.1. Materials and Reagents

*Sisymbrium officinale* L. samples were obtained from wild-growing plants collected from two locations from Croatia in May 2019: Split (43°30′43″ N 16°28′15″ E) and river Krka (43°48′17″ N 15°57′47″ E), while *Sisymbrium orientale* L. was collected in Split (43°30′26″ N 16°26′21″ E). The botanical identity of the plant material was confirmed by a local botanist, Dr. Mirko Ruščić, from the Faculty of Natural Sciences, University of Split, Croatia, and stored under voucher numbers ZOKSOF1 for the Split sample and ZOKSOR1 for the Krka river sample. Sinigrin and isopropyl isothiocyanate were obtained from Sigma-Aldrich (St. Louis, MO, USA). Progoitrin (**24*R***), epiprogoitrin (**24*S***), gluconapin (**12**), glucosinalbin (**23**), gluconasturtiin (**105**), glucobrassicin (**43**), 4-hydroxyglucobrassicin (**28**), 4-methoxyglucobrassicin (**48**), and *N*-methoxyglucobrassicin (**47**) were purchased from Phytoplan Diehm & Neuberger GmbH (Heidelberg, Germany). All other chemicals and reagents were of analytical grade. Human cancer cell lines (lung A549, bladder T24, and breast MDA-MB-231, acquired from the American Type Tissue Culture Collection (ATCC, Manassas, VA, USA)) were cultured in a humidified atmosphere with 5% CO_2_ at 37 °C in Dulbecco’s modified Eagle medium (DMEM, EuroClone, Milano, Italy) containing 4.5 g/L glucose, 10% fetal bovine serum (FBS), and 1% antibiotics (Penicillin Streptomycin, EuroClone, Milano, Italy).

### 3.2. Isolation and Chemical Analysis

#### 3.2.1. Isolation of Desulfoglucosinolates

GSLs were extracted as previously reported [26,28]. The plant material was divided into root, stem, leaf, and flower for *Sisymbrium officinale* and root, stem, and siliquae for *S. orientale*. The samples were freeze-dried using FreeZone 2.5 L freeze-dryer (Labconco, Kansas City, MO, USA) and ground to a fine powder, from which 100 mg were extracted for 5 min at 80 °C in 2 × 1 mL MeOH/H_2_O (70:30 *v*/*v*) to inactivate the endogenous myrosinase. DEAE-Sephadex A-25 anion-exchange resin (10 g, GE Healthcare) was mixed with 125 mL of ultrapure water, and the resulting mixture was stored in a refrigerator (4 °C). Each extract (1 mL) was loaded onto a mini-column filled with 0.5 mL of DEAE-Sephadex A-25 anion-exchange resin solution (1 cm height × 0.5 cm diameter) and conditioned with 25 mM acetate buffer (pH 5.6). After washing the column with 70% MeOH and 1 mL of ultrapure water, the optimal conditions for desulfation were set by adding a buffer solution. Each mini-column was loaded with 20 μL (0.35 U/mL) of purified sulfatase and left to stand for 18 h at room temperature. The desulfoGSLs were then eluted with 1.5 mL of ultra-pure H_2_O, lyophilized and diluted to 1 mL. The samples were stored at −20 °C until further analysis by HPLC-DAD-MS/MS.

#### 3.2.2. UHPLC-MS/MS Analysis

Analysis was performed on UHPLC-MS/MS (Ultimate 3000RS with TSQ Quantis MS/MS detector, Thermo Fisher Scientific, Waltham, MA, USA) using a Hypersil GOLD column (3.0 µm, 3.0 × 100 mm, Thermo Fisher Scientific, USA). A gradient consisting of solvent A (50 μM NaCl in H_2_O) and solvent B (acetonitrile:H_2_O 30:70 *v*/*v*) was applied at a flow rate of 0.5 mL/min as follows: 0.14 min 96% A and 4% B; 7.84 min 14% A and 86% B; 8.96 min 14% A and 86% B; 9.52 min 5% A and 95% B; 13.16 min 5% A and 95% B; 13.44 min 96% A and 4% B; 15.68 min 96% A and 4% B. The column temperature was held at 25 °C, and the injection volume was 5 µL. The electrospray interface was an H-ESI source operating with a capillary voltage of 3.5 kV at 350 °C. The system was operated in positive ion mode.

The amount of GSLs was quantified using a calibration curve of pure desulfosinigrin solution (ranging from 0.14 to 1.4 mM) and relative proportionality factors (RPF) for each individual desulfoGSL. The RPF values for the quantification of desulfoGSLs were as follows: RPF 1.11 for **12**, 0.50 for **23**, 1.09 for **24S** and **24*R***, 0.28 for **28**, 0.29 for **43**, 0.25 for **48**, 0.20 for **47**, 1.0 for **56**, 0.95 for **105** [29], and arbitrary RPF 1.0 for **61**, **62**.

#### 3.2.3. Isolation of Volatiles

The volatiles from the Split sample (aerial part) were isolated by two approaches: hydrodistillation (HD) and microwave-assisted distillation (MAD). Hydrodistillation was conducted in a Clevenger-type apparatus for 2.5 h using 50 g of ground aerial part (HD). A Milestone ‘ETHOS X’ microwave laboratory oven (1900 W maximum) was used for microwave-assisted distillation (MAD). A typical experiment was conducted at atmospheric pressure with 100 g of fresh plant material for 35 min at 500 W. The distillation process started after 10 min. The distillate was collected in a side-tube using a pentane trap, dried over anhydrous sodium sulfate, and stored at −20 °C, until analysis [26,28].

#### 3.2.4. GC-MS Analysis

The gas chromatography system used consisted of a gas chromatograph, model 8890 GC, equipped with an automatic liquid injector, model 7693A, and a tandem mass spectrometer (MS/MS), model 7000D GC/TQ (Agilent Inc., Santa Clara, CA, USA). The samples were analysed on a non-polar HP-5MS UI column (dimensions: 30 m length, inner diameter 0.25 mm, and stationary phase layer thickness 0.25 μm (Agilent Inc., Santa Clara, CA, USA). The column temperature program was set at 60 °C for the first 3 min and then heated to 246 °C at 3 °C/min, and maintained for 25 min isothermally. The carrier gas was helium, and the flow rate was 1 mL/min. The inlet temperature was 250 °C, and the volume of the injected sample was 1 μL. Other conditions were as follows: ionization energy was 70 eV; ion source temperature was 230 °C; the temperature of the quadrupoles was set at 150 °C. The analyses were carried out in duplicate.

The individual peaks were identified by comparison of their Kovats retention indices (relative to C_8_–C_40_ n-alkanes for HP-5MS UI column) to those from literature and/or authentic samples, as well as by comparing their mass spectra with literature from Wiley 9N08 MS (Wiley, New York, NY, USA) and NIST17 (Gaithersburg, MD, USA) mass spectral databases. The percentages in Table 2 were calculated as the mean value of component percentages on the HP-5MS UI column for analyses run in duplicate.

### 3.3. Cell Viability Assay (MTT)

An MTT spectrophotometric assay was performed on a microplate photometer, model HiPo MPP-96 (BioSan, Riga, Latvia), as previously described [26,28]. The cells were treated with Split *S. officinale* volatile isolates (HD, MAD) at concentrations of 1, 5, 10, 50, and 100 µg/mL in a complete medium (in triplicate) for 72 h. After treatment with isolated compounds, the cells were incubated with 0.5 g MTT/L at 37 °C for 2 h, the medium was removed, DMSO was added, and the mixture was incubated for another 10 min at 37 °C while shaking. The degree of formazan formation, an indicator of living and metabolically active cells, was measured at 570 nm. The data were calculated in relation to the untreated control (100%) from three independent measurements. The calculation of IC_50_ values was performed using GraphPad Prism software version 7.0 (San Diego, CA, USA), normalizing the data by three independent measurements of untreated controls. The criteria used to categorize the activity against the tested cell lines was based on IC_50_ values as follows: 20 μg/mL = highly active, 21–200 μg/mL = moderately active, 201–500 μg/mL = weakly active [30].

## 4. Conclusions

Re-investigation of *Sisymbrium officinale* by UHPLC-DAD-MS/MS, using a library of *t*_R_ and MS/MS, enabled identification of indole-type GSLs, next to the previously known branched GSLs. Met-derived GSLs were found in *S. orientale* as dominant, along with Phe and Trp ones. Previous phylogenetic investigation suggested that *S. officinale* and *S. orientale* share a common ancestor and that *S. officinale* developed much earlier. While chemical investigation suggests quite different GSL profiles, both belong to simple, ancient GSL biosynthesis, which has been suggested to lack amino acid chain elongation, tryptophan-derived GSLs, and a number of traits related to GSL biosynthesis from chain-elongated methionine. Chemical investigations of this genus are still scarce. This investigation suggests GSLs’ (bio)diversification through their evolution. However, in order to get a full picture, further chemical investigations should be conducted on other *Sisymbrium* species.

## Figures and Tables

**Table 1:** Glucosinolate (GSL) content in diverse *Sisymbrium officinale* plant parts collected from different locations.

No. *	Glucosinolate (GSL) (Trivial Name)			Glucosinolates (µmol/g DW)							
				Split				Krka			
		*t*_R_ (min)	[M + Na]^+^	Root	Stem	Leaf	Flower	Root	Stem	Leaf	Flower
	*Val-derived*										
**56**	Isopropyl GSL (glucoputranjivin)	4.12	304	1.60 ± 0.21	13.54 ± 0.35	10.62 ± 0.11	11.66 ± 0.18	tr	2.72 ± 0.05	2.88 ± 0.10	7.40 ± 0.22
	*Leu-derived*										
**62**	Isobutyl GSL	5.30	318	n.d.	tr	tr	tr	n.d.	n.d.	n.d.	tr
	*Ile-derived*										
**61**	*sec*-Butyl GSL (glucocochlearin)	5.64	318	tr	0.61 ± 0.03	tr	tr	n.d.	0.24 ± 0.06	tr	tr
	*Phe/Tyr-derived*										
**23**	4-Hydroxybenzyl GSL (glucosinalbin) ^a^	4.51	368	0.58 ± 0.08	n.d.	n.d.	n.d.	n.d.	n.d.	n.d.	n.d.
	*Trp-derived*										
**28**	4-Hydroxyindol-3-ylmethyl GSL (4-hydroxy-glucobrassicin) ^a^	5.42	407	n.d.	n.d.	tr	tr	n.d.	n.d.	1.41 ± 0.14	n.d.
**43**	Indol-3-ylmethyl GSL (glucobrassicin) ^a^	7.21	391	0.36 ± 0.04	1.70 ± 0.15	3.81 ± 0.06	3.01 ± 0.10	0.99 ± 0.13	0.74 ± 0.06	5.72 ± 0.21	4.33 ± 0.17
**48**	4-Methoxyindol-3-ylmethyl GSL (4-methoxyglucobrassicin) ^a^	8.01	421	0.46 ± 0.00	0.07 ± 0.00	n.d.	n.d.	0.77 ± 0.02	n.d.	n.d.	n.d.
**47**	*N*-Methoxyindol-3-ylmethyl GSL (neoglucobrassicin) ^a^	9.34	421	3.96 ± 0.12	n.d.	n.d.	n.d.	5.18 ± 0.18	0.02 ± 0.00	n.d.	0.04 ± 0.00
	Total (µmol/g DW)			6.96 ± 0.45	15.92 ± 1.06	14.43 ± 0.17	14.67 ± 0.28	6.94 ± 0.33	3.72 ± 0.17	10.01 ± 0.45	11.77 ± 0.39

* No.–numbers are related to the glucosinolate number given in review paper by Blažević et al. [1], and the structures are shown in Figure 1. ^a^ Compound identified by MS^2^ spectra and *t*_R_ comparison with standard. All chromatograms are given in Figure S1 (Split sample) and Figure S2 (Krka sample), while MS^2^ spectra are given in Figure S4. tr—traces; n.d.—not detected; DW—dry weight of plant material. Data are expressed as the mean value ± standard error (*n* = 3).

**Table 2:** Volatiles obtained from aerial parts of *S. officinale* from Split using different methods of isolation.

No. *	Parent Glucosinolate Identified Breakdown Compound	KI	HD (%)	MAD (%)
**56**	Glucoputranjivin			
	Isopropyl isothiocyanate ^a,b^	860	27.03	51.05
**61**	Glucocohlearin			
	*sec*-Butyl isothiocyanate ^b^	955	2.27	1.46
	*Other volatiles*			
	Nonanal ^b^	1114	tr	tr
	*β*-Cyclocitral ^b^	1231	tr	tr
	*β*-Ionone ^b^	1491	2.26	tr
	6,10,14-Trimethylpentadecan-2-one ^b^	1857	2.67	6.48
	Hexadecanoic acid ^a,b^	2027	64.59	36.33
	Total (%)		98.82	95.32
	Yield (µg/g)		24.55	8.90

* No.–numbers are related to the glucosinolate number given in review paper by Blažević et al. [1]. HD, hydrodistillation in Clevenger type apparatus; MAD, microwave-assisted distillation; KI, Kovats retention indices determined on an HP-5MS UI capillary column; tr, traces. ^a^ Compound identified by mass spectra and RI comparison with standard. ^b^ Compound identified by mass spectra comparison with Wiley/NIST library.

**Table 3:** Glucosinolate (GSL) content in *Sisymbrium orientale* from diverse plant parts.

No.*	Glucosinolate (GSL) (Trivial Name)		Glucosinolates (µmol/g DW)			
		*t*_R_ (min)	[M + Na]^+^	Root	Stem	Siliquae
	*2homoMet-derived*					
**24*R***	(2*R*)-Hydroxybut-3-enyl GSL (progoitrin) ^a^	1.41	332	tr	n.d.	3.03 ± 0.24
**24*S***	(2*S*)-Hydroxybut-3-enyl GSL (epiprogoitrin) ^a^	1.92	332	0.53 ± 0.13	0.25 ± 0.03	18.40 ± 0.22
**12**	But-3-enyl GSL (gluconapin) ^a^	4.80	316	0.73 ± 0.08	tr	0.36 ± 0.09
	*Phe/Tyr-derived*					
**23**	4-Hydroxybenzyl GSL (glucosinalbin) ^a^	4.51	368	tr	n.d.	n.d.
	*homoPhe derived*					
**105**	2-Phenylethyl GSL (gluconasturtiin) ^a^	7.93	366	7.89 ± 0.15	tr	0.06 ± 0.00
	*Trp-derived*					
**47**	*N*-Methoxyindol-3-ylmethyl GSL (neoglucobrassicin) ^a^	9.33	421	0.15 ± 0.00	n.d.	n.d.
	Total (µmol/g DW)			9.15 ± 0.36	0.25 ± 0.03	21.79 ± 0.55

* No.–numbers are related to the glucosinolate number given in review paper by Blažević et al. [1], and the structures are shown in Figure 1. ^a^ Compound identified by MS^2^ spectra and *t*_R_ comparison with standard. All chromatograms are given in Figure S3, while the MS^2^ spectra are given in Figure S4. tr–traces; n.d.–not detected; DW–dry weight of plant material. Data are expressed as the mean value ± standard error (*n* = 3). homo–higher homologue of specified amino acids.

## Data Availability

Not applicable.

## Supplementary Materials

The following are available online at https://www.mdpi.com/article/10.3390/molecules27238431/s1, Figure S1. Chromatogram of desulfoglucosinolates obtained from the different plant parts of *S. officinale* from Split: d56–desulfoisopropyl GSL (desulfoglucoputranjivin), d61–desulfo-*sec*-butyl GSL (desulfoglucocochlearin), d62–desulfoisobutyl GSL, d23–desulfo-4-hydroxybenzyl GSL (desulfoglucosinalbin), d28–desulfo-4-hydroxyindol-3-ylmethyl GSL (desulfo-4-hydroxyglucobrassicin), d43–desulfoindol-3-ylmethyl GSL (desulfoglucobrassicin), d47–desulfo-*N*-methoxyindol-3-ylmethyl GSL (desulfoneoglucobrassicin), d48–desulfo-4-methoxyindol-3-ylmethyl GSL (desulfo-4-methoxyglucobrassicin).; Figure S2. Chromatogram of desulfoglucosinolates obtained from the different plant parts of *S. officinale* from Krka: d56–desulfoisopropyl GSL (desulfoglucoputranjivin), d61–desulfo-*sec*-butyl GSL (desulfoglucocochlearin), d62–desulfoisobutyl GSL, d28–desulfo-4-hydroxyindol-3-ylmethyl GSL (desulfo-4-hydroxyglucobrassicin), d43–desulfoindol-3-ylmethyl GSL (desulfoglucobrassicin), d47–desulfo-*N*-methoxyindol-3-ylmethyl GSL (desulfoneoglucobrassicin), d48–desulfo-4-methoxyindol-3-ylmethyl GSL (desulfo-4-methoxyglucobrassicin).; Figure S3. Chromatogram of desulfoglucosinolates obtained from the different plant parts of *S. orientale*: d12–desulfobut-3-enyl GSL (desulfogluconapin), d23–desulfo-4-hydroxybenzyl GSL (desulfoglucosinalbin), d24*R*–desulfo-(2*R*)-hydroxybut-3-enyl GSL (desulfoprogoitrin), d24*S*–desulfo-(2*S*)-hydroxybut-3-enyl GSL (desulfoepiprogoitrin), d47–desulfo-*N*-methoxyindol-3-ylmethyl GSL (desulfoneoglucobrassicin), d105–desulfo-2-phenylethyl GSL (desulfogluconasturtiin).; Figure S4. MS^2^ spectra at 15V ionization of detected desulfoglucosinolates. Fragment types observed, alone or in combination, in MS^2^ spectra desulfoglucosinolates (dGSLs) in positive mode: a–Na^+^ adduct of anhydroGlc, C_6_H_10_O_5_ (at *m/z* 185) or an acyl derivative; b–Na^+^ adduct of thioGlc, C_6_H_11_O_5_SH (at *m/z* 219) or an acyl derivative; c–Loss of anhydroGlc (*m/z* 162) or an acyl derivative; d–Na^+^ adduct of Glc, C_6_H_12_O_6_ (at *m/z* 203); e–Loss of thioGlc (*m/z* 196) or an acyl derivative.

## Sample Availability

Not available.
