# Supplementary material for: Glucosinolates of Sisymbrium officinale and S. orientale
Source: Molecules. 2022 Dec 2;27(23):8431. doi: 10.3390/molecules27238431 (PMC9736730; doi:10.3390/molecules27238431)
Supplement: Supplementary file 1 [file molecules-27-08431-s001.zip › molecules-2028510-supplementary.pdf]

molecules

MDPI

Supplementary Materials

Glucosinolates of *Sisymbrium officinale* and *S. orientale*

Azra Đulović[1], Marijana Popović[1], Franko Burčul[2], Vedrana Čikeš Čulić[3], Sandra Marijan[3], Mirko Ruščić[4], Nikolina Anđelković[1], Ivica Blažević[1,*]

[1] Department of Organic Chemistry, Faculty of Chemistry and Technology, University of Split, Ruđera Boškovića 35, 21000 Split, Croatia

2 Department of Analytical Chemistry, Faculty of Chemistry and Technology, University of Split, Ruđera Boškovića 35, 21000 Split, Croatia

3 School of Medicine, University of Split, Šoltanska 2, 21000 Split, Croatia

4 Department of Biology, Faculty of Science, University of Split, Ruđera Boškovića 33, 21000 Split, Croatia

* Correspondence: blazevic@ktf-split.hr; Tel.:+385 21 329 434

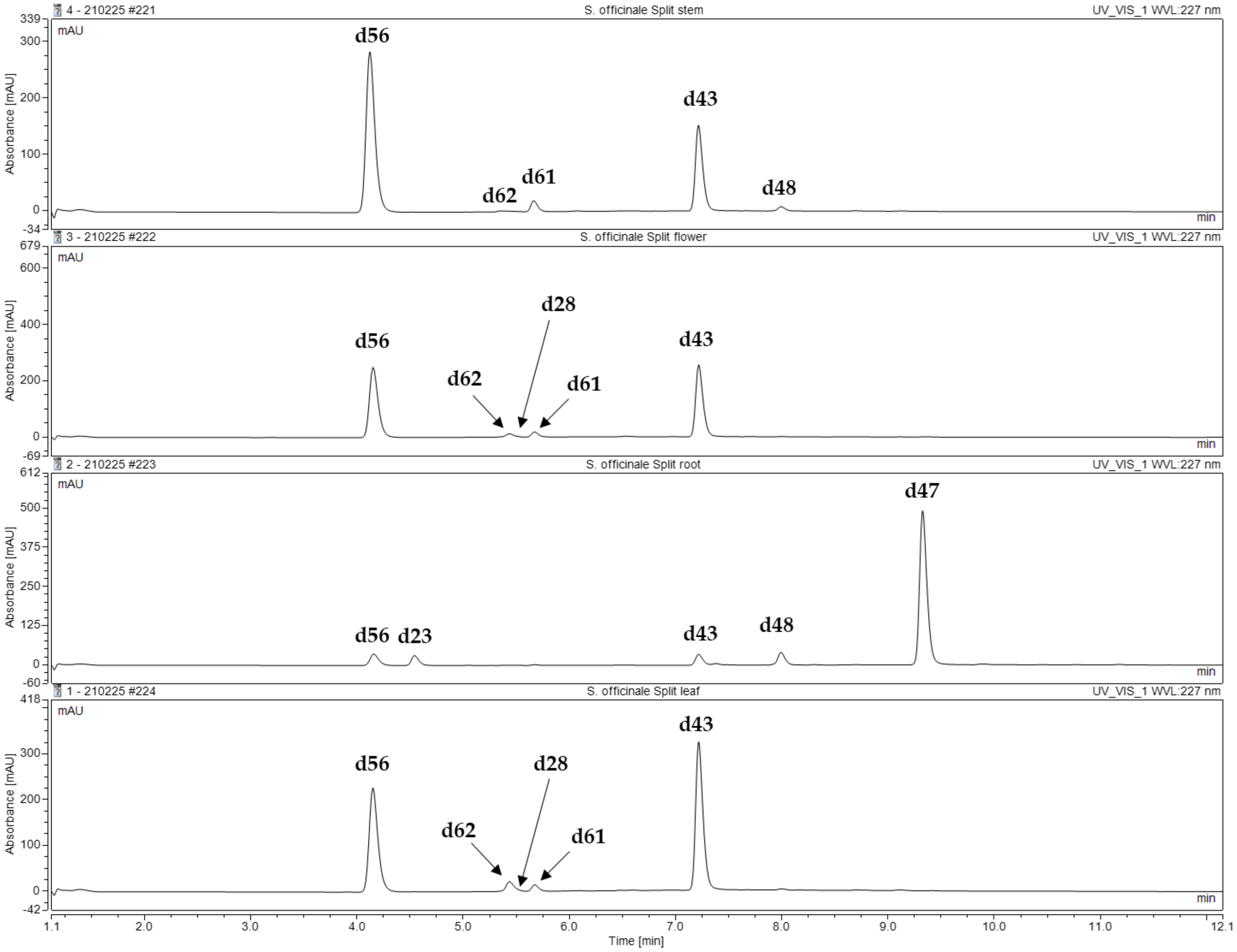

Figure S1. Chromatogram of desulfoglucosinolates obtained from the different plant parts of *S. officinale* from Split: **d56** - desulfoisopropyl GSL (desulfoglucoputranjivin), **d61** - desulfo-*sec*-butyl GSL (desulfoglucocochlearin), **d62** - desulfoisobutyl GSL, **d23** - desulfo-4-hydroxybenzyl GSL (desulfoglucosinalbin), **d28** - desulfo-4-hydroxyindol-3-ylmethyl GSL (desulfo-4-hydroxyglucobrassicin), **d43** - desulfoindol-3-ylmethyl GSL (desulfoglucobrassicin), **d47** - desulfo-*N*-methoxyindol-3-ylmethyl GSL (desulfoneoglucobrassicin), **d48** - desulfo-4-methoxyindol-3-ylmethyl GSL (desulfo-4-methoxyglucobrassicin).

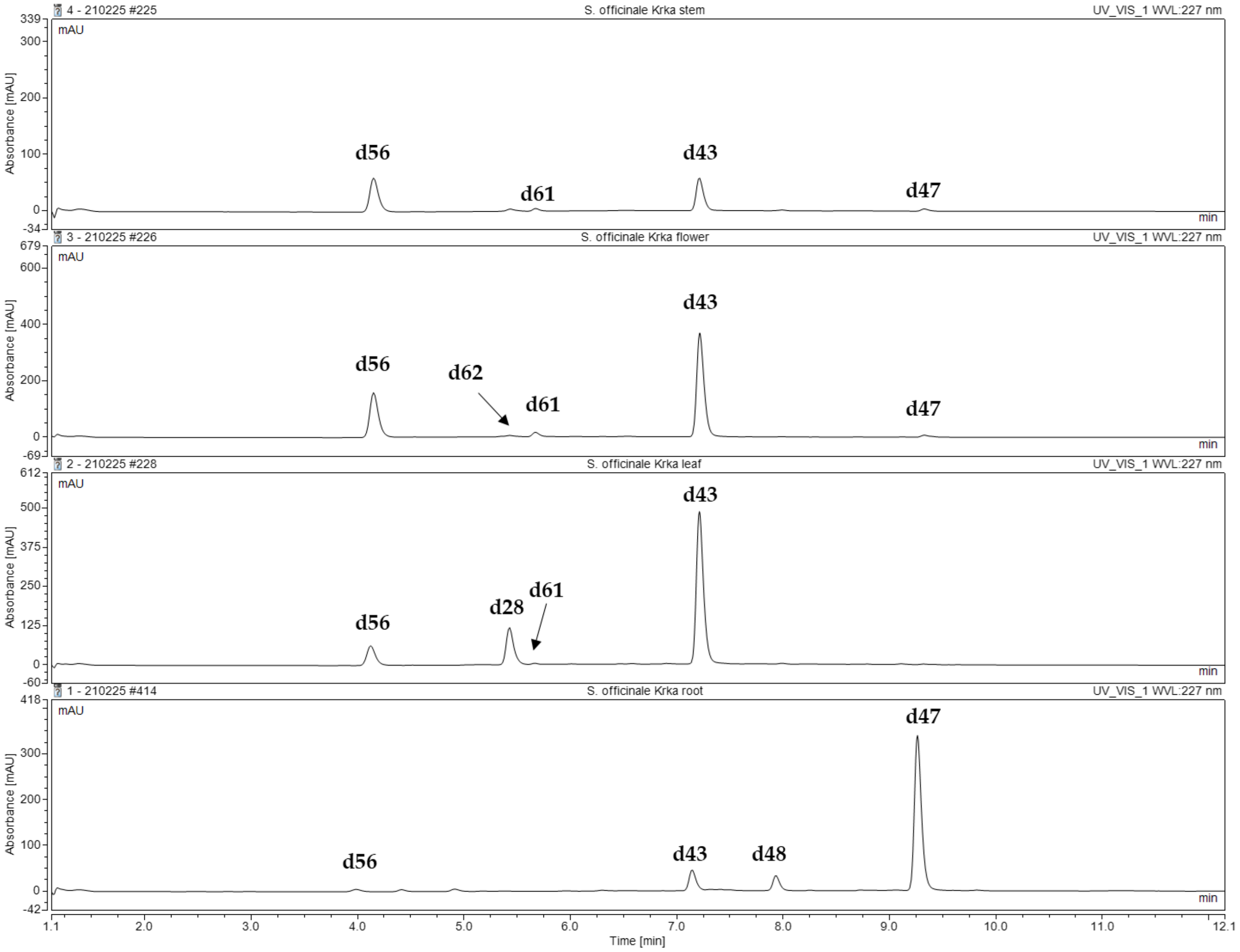

Figure S2. Chromatogram of desulfoglucosinolates obtained from the different plant parts of *S. officinale* from Krka: **d56** - desulfoisopropyl GSL (desulfoglucoputranjivin), **d61** - desulfo-*sec*-butyl GSL (desulfoglucocochlearin), **d62** - desulfoisobutyl GSL, **d28** - desulfo-4-hydroxyindol-3-ylmethyl GSL (desulfo-4-hydroxyglucobrassicin), **d43** - desulfoindol-3-ylmethyl GSL (desulfoglucobrassicin), **d47** - desulfo-*N*-methoxyindol-3-ylmethyl GSL (desulfoneoglucobrassicin), **d48** - desulfo-4-methoxyindol-3-ylmethyl GSL (desulfo-4-methoxyglucobrassicin).

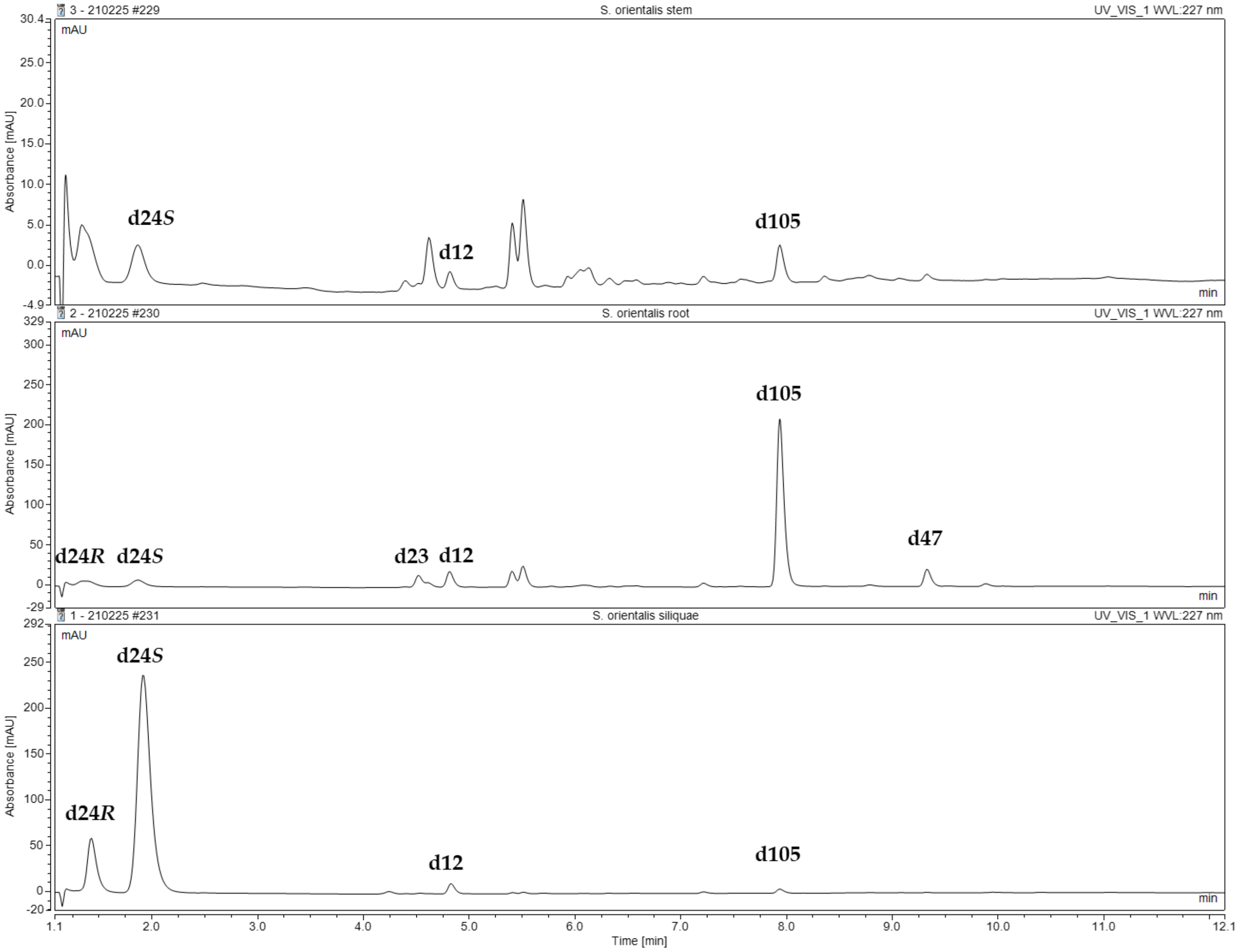

Figure S3. Chromatogram of desulfoglucosinolates obtained from the different plant parts of *S. orientale*:

d12 - desulfobut-3-enyl GSL (desulfogluconapin), **d24*R*** - desulfo-(2*R*)-hydroxybut-3-enyl GSL (desulfoprogoitrin), **d24*S*** - desulfo-(2*S*)-hydroxybut-3-enyl GSL (desulfoepiprogoitrin), , **d23** - desulfo-4-hydroxybenzyl GSL (desulfoglucosinalbin), **d47** - desulfo-*N*-methoxyindol-3-ylmethyl GSL (desulfoneoglucobrassicin), **d105** - desulfo-2-phenylethyl GSL (desulfogluconasturtiin).

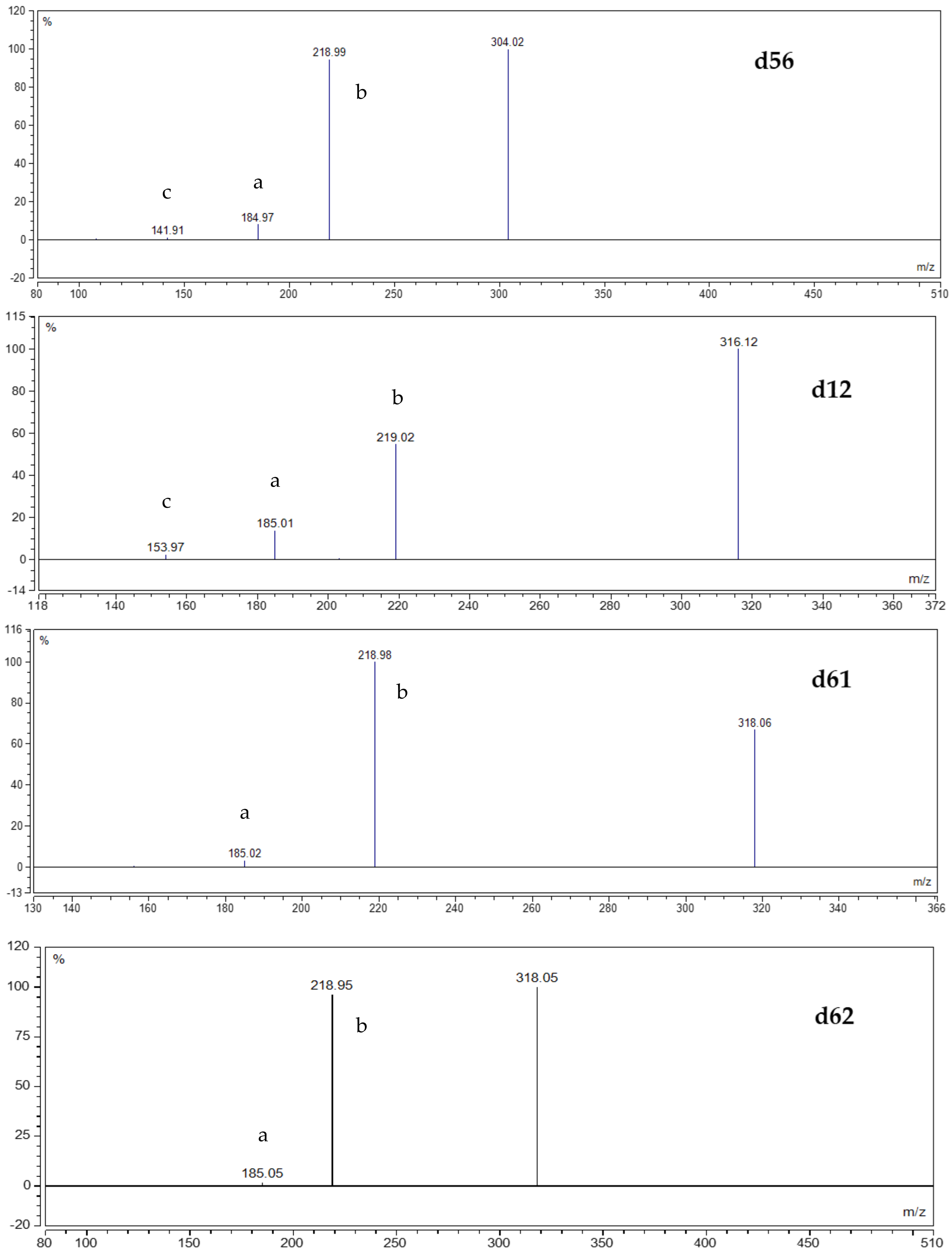

Figure S4. *Continuous*

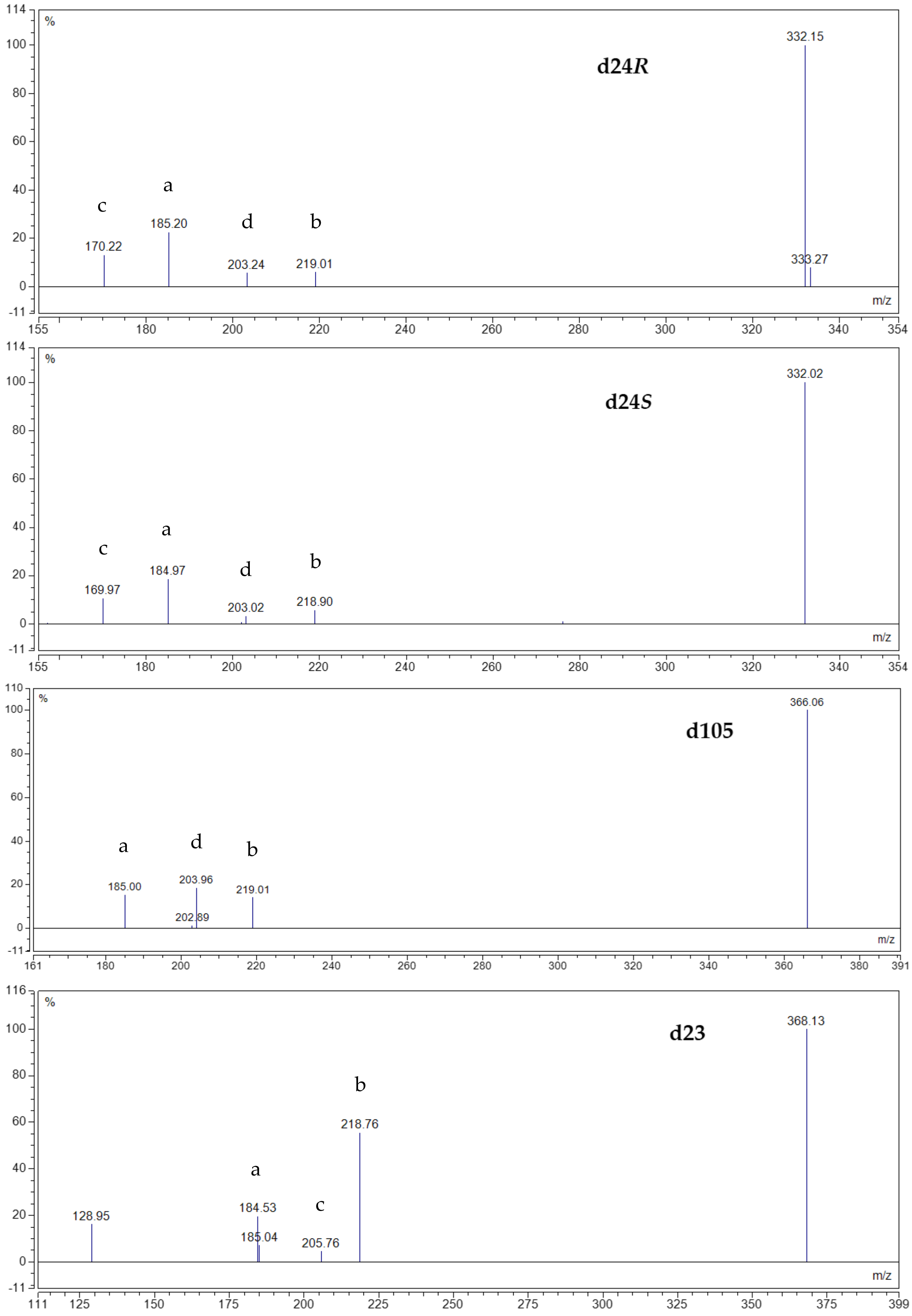

Figure S4. *Continuous*

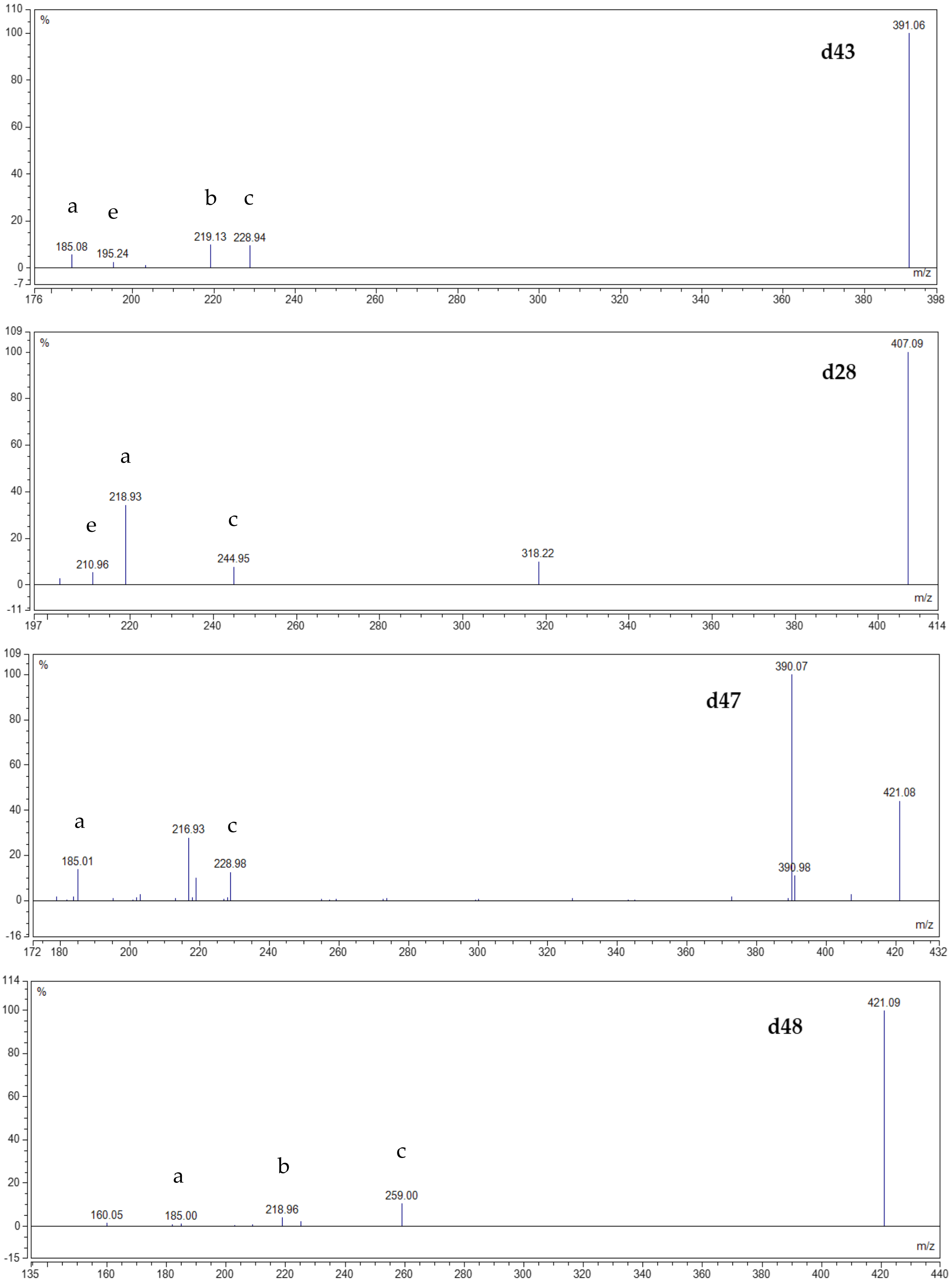

Figure S4. MS[2] spectra at 15V ionization of detected desulfoglucosinolates. Fragment types observed, alone or in combination, in MS2 spectra desulfoglucosinolates (dGSLs) in positive mode: a - Na^+ adduct of anhydroGlc, $C_6H_{10}O_5$ (at m/z 185) or an acyl derivative; b - Na^+ adduct of thioGlc, $C_6H_{11}O_5SH$ (at m/z 219) or an acyl derivative; c - Loss of anhydroGlc (m/z 162) or an acyl derivative; d - Na^+ adduct of Glc, $C_6H_{12}O_6$ (at m/z 203); e - Loss of thioGlc (m/z 196) or an acyl derivative.
